# Coencapsulation of ISCs and MSCs Enhances Viability and Function of both Cell Types for Improved Wound Healing

**DOI:** 10.1007/s12195-019-00582-3

**Published:** 2019-07-01

**Authors:** Ayesha Aijaz, Matthew Teryek, Michael Goedken, Marianne Polunas, Ronke M. Olabisi

**Affiliations:** 1 Department of Biomedical Engineering, Rutgers University, 599 Taylor Road, Piscataway, NJ 08854 USA; 2 Research Pathology Services, Rutgers University, Piscataway, NJ 08854 USA

**Keywords:** Mesenchymal stem cells, Insulin, Microencapsulation, Cell encapsulation, Cell therapy, Wound healing

## Abstract

**Introduction:**

We previously demonstrated that insulin secreting cells (ISCs) accelerate healing of chronic wounds, and it is known that mesenchymal stem cells (MSCs) also accelerate wound healing. Here, we report that the combination of both cell types coencapsulated into a synthetic hydrogel dressing accelerates chronic wound healing 3 × faster than control and 2 × faster than each cell type delivered singly. Specifically, insulin released by ISCs activates the PI3/Akt pathway, which is vital to the function and survival of MSCs. MSCs in turn improve the viability and function of ISCs.

**Materials and Methods:**

MSCs and/or rat islet tumor RIN-m cells were encapsulated into polyethylene glycol diacrylate hydrogel sheets and applied to 1 cm^2^ full thickness excisional wounds on the dorsa of genetically diabetic male mice (BKS.Cg-m +/+Leprdb/J) in accordance with protocols approved by the Rutgers IACUC. Encapsulated cell viability was assessed using a LIVE/DEAD^®^ Viability/Cytotoxicity Kit. Akt phosphorylation, insulin, VEGF, and TGF-β1 secretion were assessed by ELISA. Animals were sacrificed on postoperative days 14 and 28 and wound tissue was collected for histological and western blot analysis.

**Results:**

ISC:MSC combination groups had the highest levels of every secreted product and phosphorylated Akt, and closed wounds in 14 days, ISC-only or MSC-only groups closed wounds in 28 days, control groups closed wounds in 40 days. Further, ISC:MSC groups healed without intermediate scab or scar.

**Conclusions:**

Combining MSCs with ISCs results in a more robust healing response than singly delivered cells, warranting further investigation of coencapsulation for MSC therapies.

## Introduction

Skin provides thermoregulation and sensory information, assists with fluid homeostasis, and acts as a barrier to infection.^7^ Chronic wounds upset skin function, resulting in morbidities and mortalities that increase with healing time. One veterans affairs hospital reported that the pivotal event leading to amputation in 73% of its amputees was faulty wound healing.^26^ Chronic wounds are skin injuries that recur or fail to heal by 6 weeks. These wounds affect 6.5 million patients in the US and this number is growing rapidly due to an aging population and the drastic increase in the numbers of diabetics, the obese, and the elderly; all populations in which wound healing is impaired.^31^ Chronic wounds also affect those with mobility impairments. Prolonged immobility results in an increased incidence of pressure ulcers, which are immobility-related chronic wounds affecting both skin and underlying tissue.

Endogenous mesenchymal stem cells (MSCs) coordinate the normal healing response by regulating immune and inflammatory responses, secreting growth factors and matrix proteins, and recruiting other necessary host cells. In chronic wounds, this process is stalled and exogenous MSCs have been remarkably successful restarting it.^18^,^25^,^37^ Unlike traditional topical ointments where dosage is controlled by application frequency, MSCs applied to wounds alter their secreted biofactors in response to their environment. MSCs secrete growth factors that reduce inflammation and increase angiogenesis, collagen synthesis, and macrophage and fibroblast migration.^5^,^24^ Insulin delivery to chronic wounds is also a powerful healing agent.^23^,^28^ Insulin accelerates wound healing by activating pathways that stimulate the migration and differentiation of skin cells such as keratinocytes.^3^

The challenge in supplying wounds with both insulin and MSCs has been a delivery system capable of supporting MSCs while supplying a sustained, steady dose of fresh insulin, which has a short half-life.^39^ Though a variety of topical insulin preparations are available, they have not been designed to support MSCs. Typically, these preparations consist of crystal insulin dissolved within a carrier.^3^,^28^ They do not contain the cell nutrients typically found in culture media, nor the serum proteins, and many applications are in liquid form, which would not localize the MSCs to the wound site. Further, such topical preparations require repeated applications that would disturb exogenous MSCs. And finally, these preparations do not overcome the difficulties in supplying sufficient and persistent insulin levels at the wound site, a challenge that is thought to reduce insulin’s wound healing effectiveness.^39^ Here, we overcome these challenges by encapsulating MSCs and insulin secreting cells (ISCs) within a highly bioinert synthetic hydrogel. In this way ISCs are employed as factories for insulin delivery, and the hydrogel prevents MSC migration and provides immunoprotection for both cell types. The hydrogel absorbs and retains wound fluid, which supplies the cells with nutrients and permits waste exchange. Our results have confirmed findings that MSCs improve ISC function and survival.^6^,^36^ Interestingly, our results further suggest that ISCs also improve MSC function and survival. Though the latter result has not been directly established in the literature, it is supported by research over the past decade demonstrating that phosphatidylinositol 3′-kinase—anti-serine-threonine kinase/protein kinase B (PI3K–Akt/PKB) plays a key role in the signal transduction pathways that are activated by insulin and is believed to contribute to several cellular functions including transcriptional regulation and cell survival.^33^ Our results showed both cell types releasing more growth factors and insulin when combined than when separate, and the combination of cells accelerated wound closure much faster than when cells were applied separately. Based on our exciting results we believe the combination of ISCs and MSCs will be the next generation of wound healing agents.

## Materials and Methods

Unless otherwise indicated, reagents were purchased from MilliporeSigma (St. Louis, MO) and tissue culture incubators were humidified incubators maintained at 37 °C with 5% CO_2_.

### Cell Culture

Rat insulinoma beta RIN-m cells were purchased from American Type Culture Collection (ATCC, Manassas, VA) and propagated in RPMI-1640 medium (ATCC) supplemented with 10% fetal bovine serum (FBS) and 1% w/v penicillin–streptomycin (pen–strep) in a tissue culture incubator. Human bone-marrow derived MSCs were provided by the Texas A&M Health Science Center College of Medicine Institute for Regenerative Medicine at Scott & White through a grant from ORIP of the NIH, Grant # P40OD011050 at passage 1 and propagated in alpha-minimal essential medium (α-MEM) without deoxyribo- or ribo-nucleotides, supplemented with 10% v/v FBS (Atlanta Biologicals, Flowery Branch, GA), 1% w/v pen-strep, 4 mM l-glutamine (Life Technologies) and 1 ng/mL basic fibroblast growth factor (bFGF; Life Technologies).

### Cell Encapsulation

RIN-m cells (ISCs) and/or human MSCs (hMSCs) were encapsulated within PEGDA hydrogel sheets by photopolymerizing cells suspended in a precursor solution formed by combining 0.1 g/mL 10 kDa PEGDA (10% w/v; Laysan Bio, Inc.) with (1.5% v/v) triethanolamine/HEPES buffered saline (pH 7.4), 37 mM 1-vinyl-2-pyrrolidinone, 0.1 mM eosin Y. The prepolymer solution was combined with cells (1 × 10^4^ cells/μL), pipetted into 1 cm^2^ custom made molds and exposed to white light for 20 s to achieve 400 *µ*m thick cell-laden hydrogel sheets containing both MSCs and ISCs, MSCs, or ISCs at 0.5 × 10^6^ cells per 400 *µ*m thick hydrogel sheets. Crosslinked hydrogels were gently lifted with blunt forceps and placed in Transwells^®^ (0.4 mm pore polycarbonate membrane Transwell^®^ inserts; Corning, Inc., Lowell, MA) in a 12-well plate with 3 mL culture medium. Hydrogels were maintained in a tissue culture incubator. For monolayer comparisons, 1 × 10^6^ ISCs or hMSCs were plated on 6-well tissue culture plates.

### Cell Viability

Encapsulated cell viability was assessed on days 1, 7 and 21 by calcein acetoxymethyl ester/ethidium homodimer fluorescent stain as described previously.^1^ Briefly, hydrogels were submerged in media, 2 mM calcein acetoxymethyl ester, and 4 mM ethidium homodimer (LIVE/DEAD^®^ Viability/Cytotoxicity Kit for mammalian cells; Life Technologies, Grand Island, NY). Viable cells within the hydrogel sheets were identified based on intracellular enzymatic conversion of non-fluorescent calcein-AM to green fluorescent calcein while ethidium homodimer-1 penetrated damaged membranes of dead cells and upon binding to nucleic acids fluoresced bright red. After 10 min in a tissue culture incubator, each hydrogel was imaged under an epifluorescent microscope (Zeiss Axiovert Observer Z1) and 10–15 optical slices at 28 *µ*m intervals were taken in the z-plane at three diagonal positions (*e.g.*, top left, center, and bottom right) in the x–y coordinates. Green fluorescent images for live cells and red fluorescent images for dead cells for each optical slice were separately processed on ImageJ software (Rasband, National Institutes of Health) to obtain cell counts.

### Insulin and MSC Factor Detection

Singly encapsulated cells and ISCs coencapsulated with MSCs in PEGDA hydrogel sheets were maintained in complete culture medium in tissue culture incubators. Conditioned media (CM) containing insulin and/or MSC factors released from the cell-laden hydrogel sheets were collected on days 1, 7 and 21. Media were changed on every second day, such that odd days had 24 h of insulin and/or MSC factor accumulation. Concentrations of insulin and the MSC factors TGF-β1 and VEGF in the CM were assessed by ELISA.

### Keratinocyte Scratch Assay

Scratch assays were conducted with human keratinocytes (HaCaT; Addex Bio, San Diego, CA) as previously described.^1^ Briefly, HaCaT cells were propagated in DMEM, supplemented with 10% FBS, pen-strep in tissue culture incubators. For scratch assays, 180,000 HaCaT cells/well were seeded onto 24 well plates (Greiner Bio-one; Monroe, NC). Cells were cultured for 48 h to form a confluent monolayer at which time a single scratch of 415 *µ*m ± 73 *µ*m was produced in the center of the well with a 10 *µ*L pipette tip. The wells were washed twice with Dulbecco’s Phosphate Buffered Saline (DPBS) to remove cell debris. HaCaT cells were then stimulated with CM containing insulin and/or MSC factors. CM was sampled from positive control monolayers on day 1 or cell-laden hydrogels on days 1, 7 and 21. Media from empty hydrogels was used as negative controls. HaCaT cell migration across the scratch was imaged at 0, 24, 48, 72 and 96 h using phase contrast microscopy and analyzed on NIH ImageJ software.

### Akt Phosphorylation

Rat L6 myoblast cells (ATCC) were plated at 112,500 cells/mL in a 96 well plate with 300 *µ*L per well. L6 myoblasts are known to produce phosphorylated Akt (p-Akt) when stimulated by numerous wound healing moieties. L6 myoblasts were stimulated with CM from release experiments and the levels of p-Akt were compared against the levels of total-Akt (T-Akt) using a Fast Activated Cell-based ELISA kit.

### Wound Healing Studies

All animal procedures were performed on genetically diabetic male mice (BKS.Cg-m +/+Leprdb/J; 10 weeks old, Jackson Laboratories, Bar Harbor, ME) in accordance with protocols approved by the Rutgers University institutional animal care and use committee (IACUC) as previously described.^1^ Briefly, 24 mice were divided into 4 groups (n = 6 per group): (1) phosphate buffered saline (PBS, 50 *µ*L); or hydrogels containing (2) ISCs; (3) MSCs; or both (4) ISC:MSC. Mice were further divided into mice sacrificed on day 14 and on day 28, n = 12 per time point. Thus, for days 1–14, each group had 6 animals and after sacrifice on day 14, each group had 3 animals. The four treatments were applied to full thickness 1 cm × 1 cm excise wounds on the dorsa of the mice. Hydrogels were then covered with Tegaderm™ and photographed at postoperative days 3, 7, 14, 18, 21, and 28 for gross appearance. Wound area was measured using the variance setting of the NIH ImageJ MRI Wound Healing Tool. Wound closure was calculated as percent area of original wound. Mice were weighed on the day of surgery, then weekly until sacrifice. At sacrifice blood was drawn from the tail vein and blood glucose levels were measured with a blood glucose monitoring kit (ACCU-CHEK Aviva Plus; Roche, Basel, Switzerland). Animals were sacrificed by CO_2_ inhalation and wound tissue was collected for histology.

### Histological Analysis

After sacrifice, wound tissue was explanted, fixed in 10% formalin for 24 h at room temperature, then stored in 70% ethanol at 4 °C until serial sectioning (5 *μ*m thick). Then wounds were sectioned and stained with hematoxylin and eosin (H & E) for morphological analysis, picric acid sirius red (PASR) for collagen fiber density, Ki67 for cell proliferation, alpha smooth muscle actin (α-SMA) for wound contraction, and CD31 for neovascularization at the Digital Imaging and Histology Core, Rutgers-NJMS Cancer Center. Stained slides were given to a veterinary pathologist, who scored slides for gross appearance and presence of ulcers. Histological images were analyzed on ImageJ software to determine epidermal and dermal thickness and collagen density. RGB images of PASR-stained sections were converted to gray scale by splitting the images into red, green and blue channels. Thresholded images were analyzed to measure percent area of collagen density.^10^ PASR stained histology slides were also imaged under cross-polars for collagen birefringence.

### Statistical Analysis

All data were taken in triplicate and reported as mean ± standard deviation in the text while error bars in graphs show standard error. Different treatment groups were compared using a one-way analysis of variance (ANOVA). Pairwise comparisons were made between groups using Fisher’s Least Significant Difference (LSD) post hoc test. *p*-values less than 0.05 were considered significant. All analyses were performed using KaleidaGraph statistical software version 4.1.0, Synergy Software (Reading, PA).

## Results

### Encapsulated Cell Viability and Function

**Figure 1:**
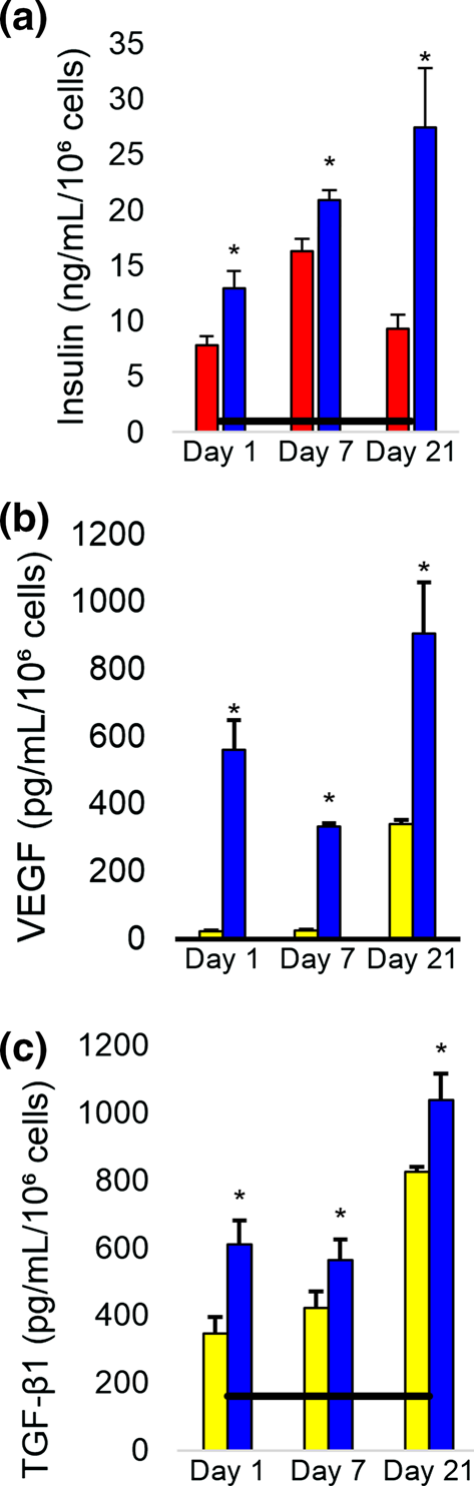
Biofactor release over time. (**a**) Insulin, (**b**) VEGF, and (**c**) TGF-β1 levels, respectively, released in the media as measured by ELISA. ISC:MSC groups had the highest levels of every secreted product and healed wounds fastest. Asterisks indicate statistically significant differences (*p* < 0.05) between hydrogel encapsulated cells vs. singly encapsulated cells. All groups were statistically greater than monolayer controls. Horizontal black bar shows monolayer controls. Error bars show standard deviation. Yellow filled square MSCs, red filled square ISCs, blue filled square ISC:MSC.

Fluorescent assays were used to assess cell viability and over the course of 21 days cell viability was highest on day 1 at 82.6 ± 1.3%, and dropped to 43.9 ± 4.2% on day 21. Coencapsulation improved biofactor release as determined by ELISA assays. The ISC:MSC dual-cell hydrogels that healed wounds faster were shown to release more insulin, vascular endothelial growth factor (VEGF), and transforming growth factor β1 (TGF-β1) than hydrogels containing singly encapsulated ISCs or MSCs (Fig. 1). Over the course of the 3 week *in vitro* study, insulin secretion levels from ISC-only hydrogels ranged between 7.8 and 16.3 ng/mL/10^6^ cells. When ISCs were combined with MSCs, these values increased to 12.9–27.5 ng/mL/10^6^ cells. Similarly, coencapsulation with ISCs improved growth factor release from MSCs. In MSC-only hydrogels, secreted TGF-β1 levels ranged between 347.1 and 824.6 pg/mL/10^6^ cells and when combined with ISCs, these levels increased to 610.4–1038.4 pg/mL/10^6^ cells. TGF-β1 levels secreted from monolayer MSCs reached only 161.6 ± 102.3 pg/mL/10^6^ cells, suggesting that encapsulation in and of itself stimulated TGF-β1 release. Likewise, VEGF release was not detected in MSC monolayers, but was detected in MSC and ISC:MSC hydrogels. VEGF release was also increased when MSCs were coencapsulated with MSCs, from 23.6 to 341.8 pg/mL/10^6^ cells when MSCs were alone to 334.9–907.7 pg/mL/10^6^ cells when cells were coencapsulated.

### Bioactivity of Released Products

**Figure 2:**
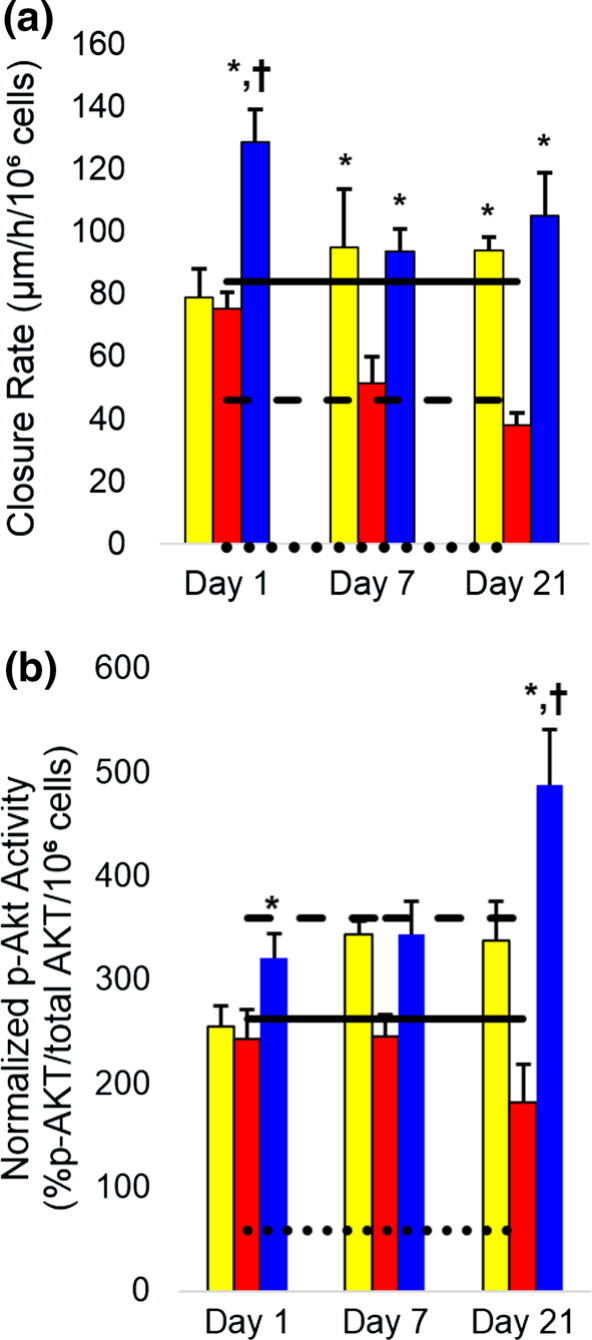
Bioactivity of insulin and MSC factors. (**a**) The closure rate of scratches in keratinocyte monolayers; (**b**) Akt phosphorylation of L6 myoblasts. Dotted lines indicate empty hydrogel controls, dashed lines are ISC monolayers, and solid lines represent MSC monolayers. All groups were statistically greater than control. Asterisks indicate statistically significant increases (*p* < 0.05) compared to other hydrogel groups; crosses indicate significant increases (*p* < 0.05) compared to all groups. All cell-laden hydrogels promoted significant HaCaT migration and Akt phosphorylation compared to control. Error bars show standard error of mean. Yellow filled square MSCs, red filled square ISCs, blue filled square ISC:MSC.

After confirming the presence of insulin, TGF-β1, and/or VEGF, the bioactivity of these biofactors was evaluated. Keratinocyte scratch assays were conducted to evaluate the biofactors’ ability to promote keratinocyte migration. The fastest scratch closure rates were observed with ISC:MSC hydrogels, which were significantly faster than either MSC or ISC hydrogels; the latter two were not statistically distinct from each other (Fig. 2). All treated scratches in keratinocyte monolayers closed significantly faster than control—ISC:MSC, MSC, and ISC hydrogels closed scratches at rates of 128.6 ± 10.53, 79.02 ± 8.9, and 75.1 ± 6.61 *µ*m/h/10^6^ cells, respectively, compared to the control rate of − 1.2 ± 1.7 *µ*m/h/10^6^ cells. The scratch closure rates of keratinocytes treated with ISC hydrogels slowed over time while ISC:MSC and MSC-treated scratches’ closure rates did not.

In addition to scratch assays, factor bioactivity was assessed by measuring the levels of phosphorylated anti-serine-threonine kinase (p-Akt) produced when L6 myoblasts were exposed to conditioned media from the hydrogels (Fig. 2b). L6 myoblasts are known to produce p-Akt when stimulated by insulin or MSCs. Akt phosphorylation provides a measure of the bioactivity of insulin and MSC growth factors since both are known to activate the Akt pathway.^4^,^14^ All cell-laden hydrogels stimulated significant Akt phosphorylation compared to controls; no significant decrease in the levels was observed for the 3-week duration. Phosphorylated Akt levels increased with increasing insulin release and that induced by ISC:MSC was significantly higher for 21 days compared to all other hydrogel constructs.

### *In Vivo* Wound Healing Response

**Figure 3:**
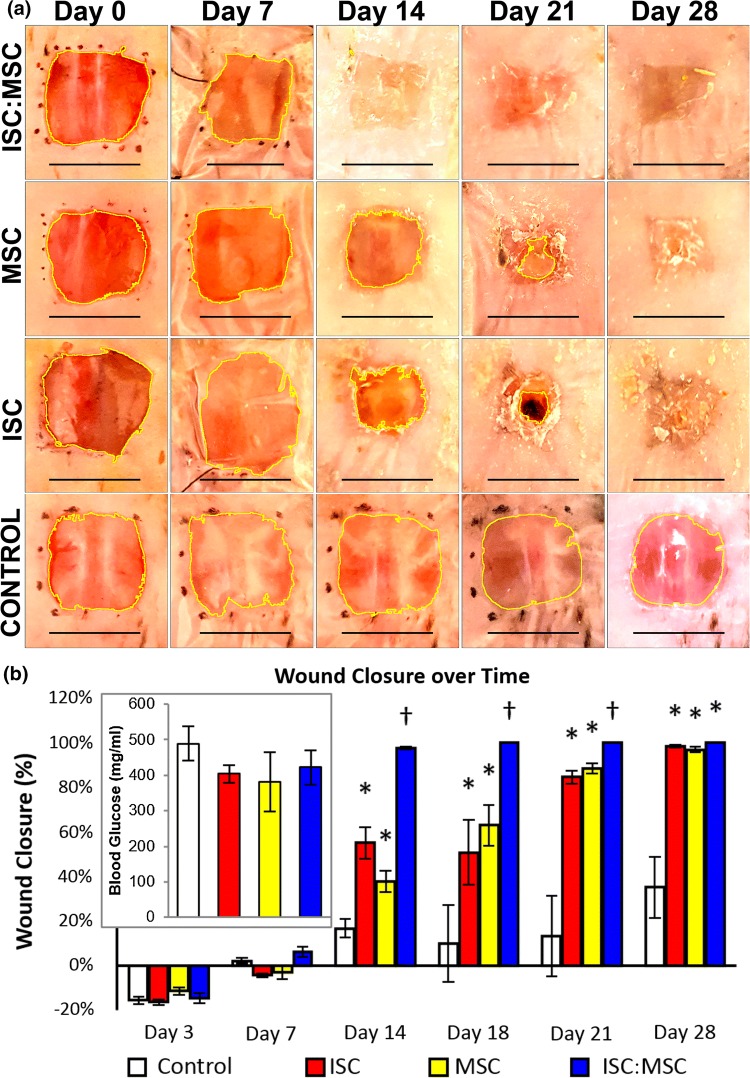
Progression of wound healing. (**a**) Rows follow single animals. After Day 0, unhealed wounds are imaged through dressings. Day 14 ISC:MSC shows closed wounds with dried hydrogel residue. Images were uniformly adjusted for contrast, saturation, and brightness. Percent wound closure and glucose levels (inset). Asterisks indicate statistically significant increases (*p* < 0.05) compared to control; crosses represent significance compared to all other groups. Error bars show standard error of mean.

Wounds that were treated with both cell types (ISC:MSCs) completely closed 5 of 6 dorsal excise wounds in genetically diabetic mice by day 14, with the 6th wound closed by day 18 (Fig. 3). Percent wound closure was quantified by measuring wound area over time using NIH ImageJ’s MRI Wound Healing tool. Early wound areas are negative (Fig. 3b) due to inherent elasticity of the skin causing tension on wounds. No external tension was applied to wounds. No scab formation was observed in any ISC:MSC-treated animals. New skin in these animals was soft to the touch, while all other mice had either open wounds or hard scabs. Wounds that were treated with single cells (ISCs or MSCs) did not close until day 28. Control wounds not treated with cells failed to close by sacrifice on day 28. Throughout the study, animal weight remained between 40 and 45 mg and blood glucose levels of all mice remained at levels indicative of diabetes (~ 400 mg/dL).

### Histological Analysis

**Figure 4:**
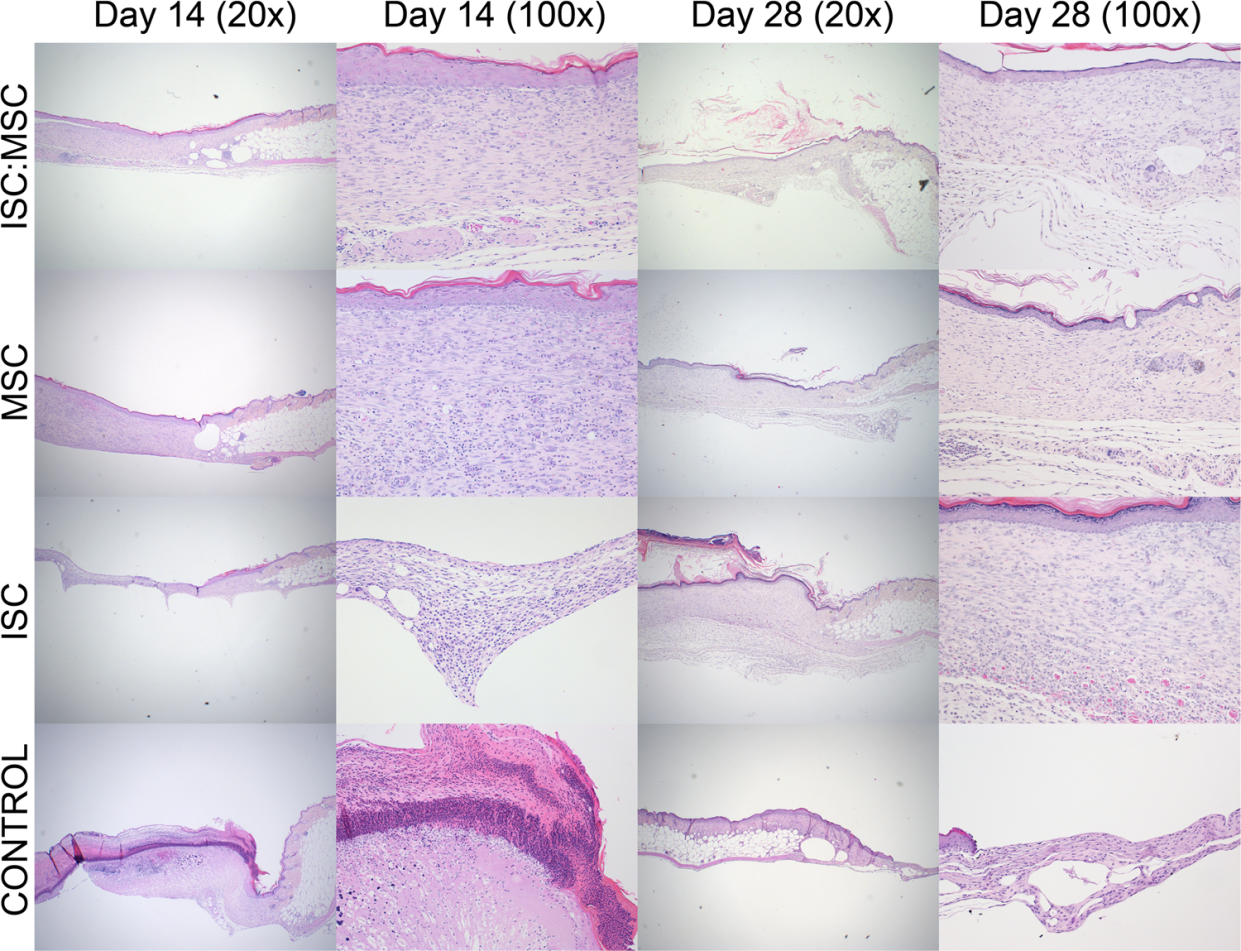
Day 14 and 28 H&E stained sections of treatment groups and untreated control wounds at two magnifications. Images were uniformly adjusted for contrast, saturation, and brightness. For each × 20 image in the panel, the left field of view shows healed/healing skin, while the right third shows uninjured skin. Each × 100 image shows healed/healing skin. × 20 refers to × 10 magnification at the objective and × 2 at the eyepiece while × 100 refers to × 10 magnification at the objective and × 10 at the eyepiece.

**Figure 5:**
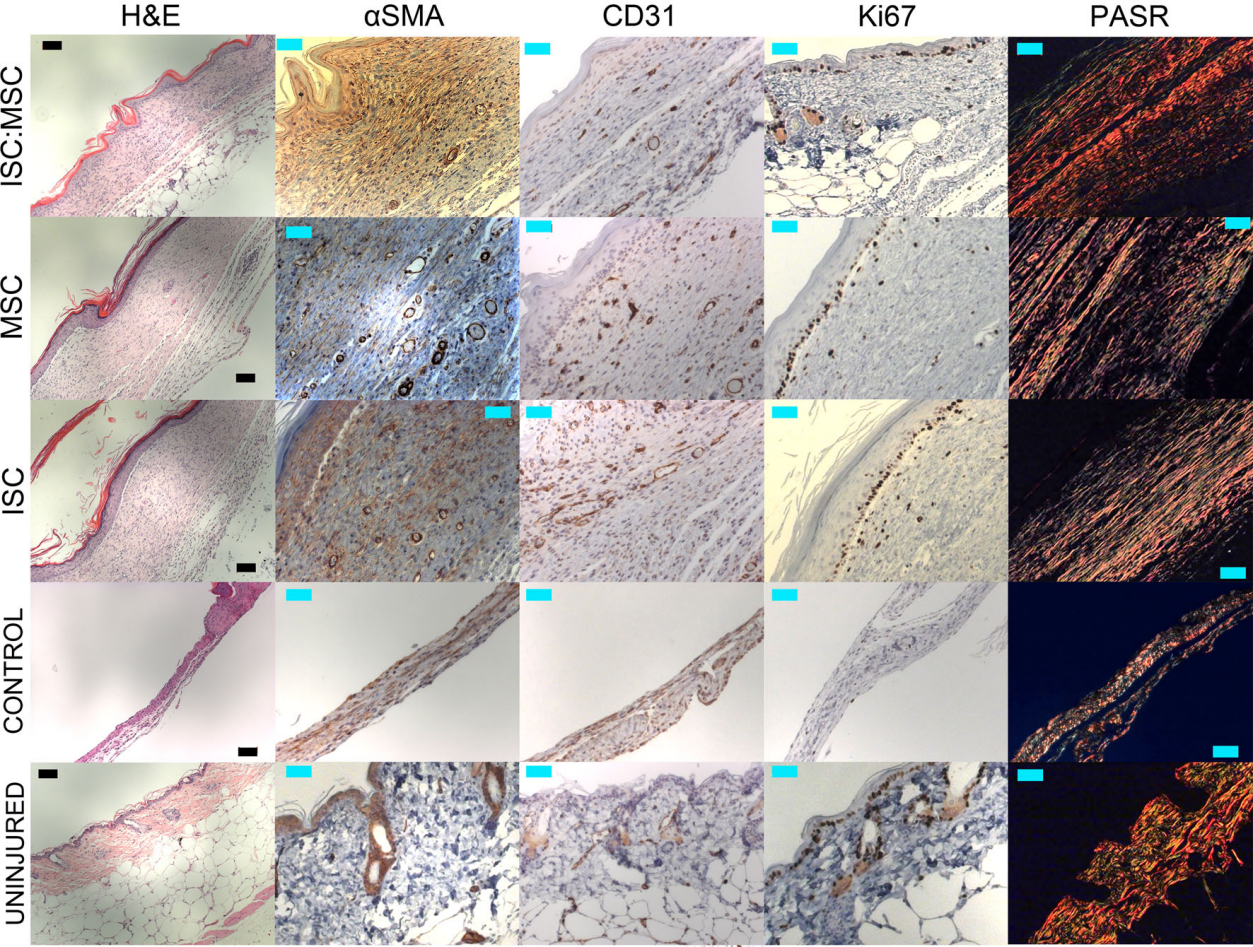
Day 28 H&E, αSMA, CD31, Ki67, and PASR stained sections of treatment groups, untreated control wounds, and uninjured skin. Sections are all within healing areas. Black scale bars are 100 *µ*m; cyan scale bars are 50 *µ*m. Most notable features are the return of a hypodermal layer and the increased level of collagen I (PASR, orange) compared to collagen III (PASR, green) in ISC:MSC-treated skin.

**Figure 6:**
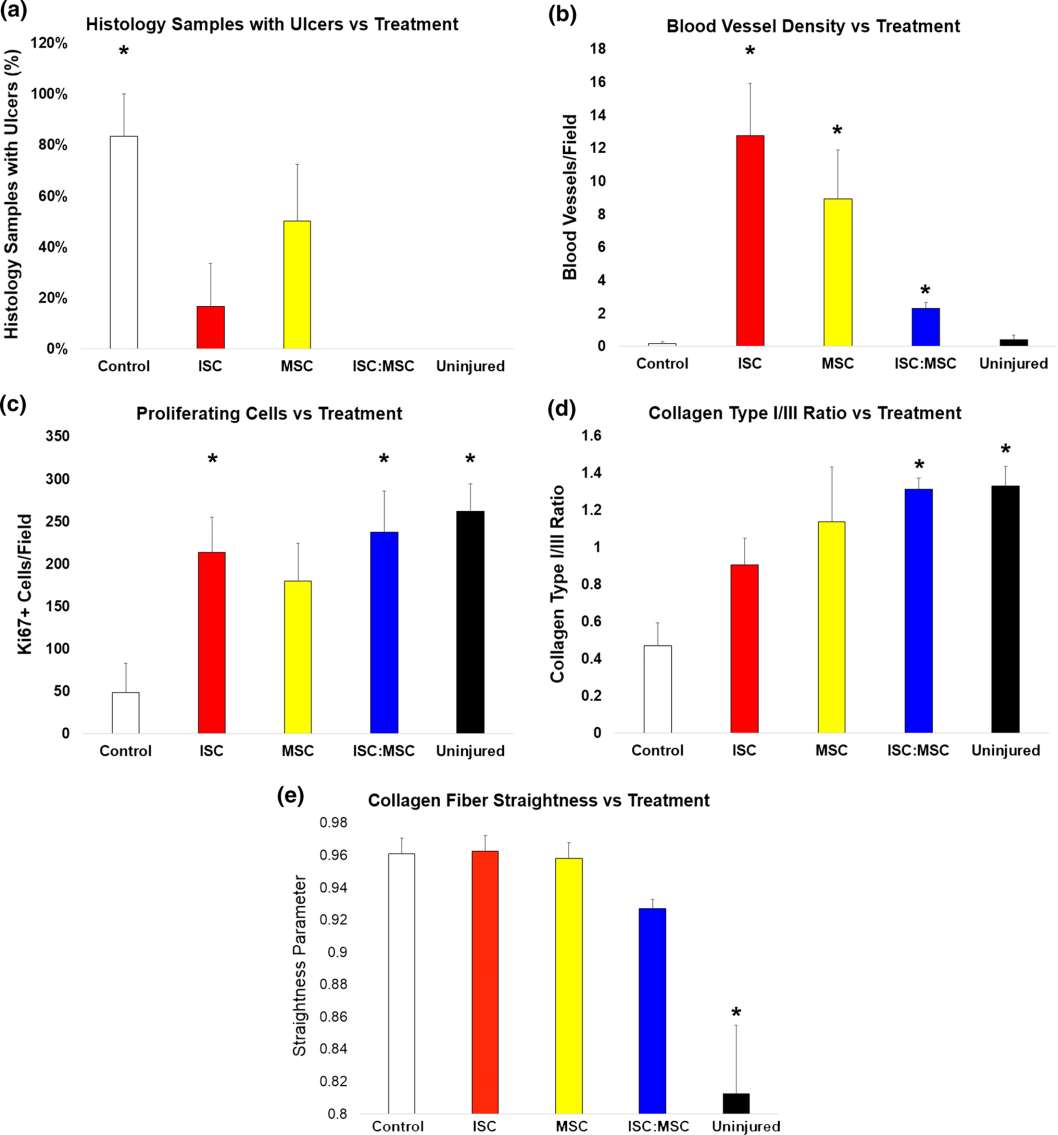
Quantitated histology features from day 28 slides. (**a**) Sections were scored for presence or absence of ulcers and tabulated. Both uninjured and ISC:MSC had none. (**b**) CD31 positive blood vessels were counted as a measure of neovascularization, which is absent in newly wounded tissues, increases during healing, then decreases in mature skin. (**c**) Ki67 positive cells per field were counted as a measure of proliferating cells. (**d**) Polarized PASR sections were used to determine relative collagen I/III content. (**e**) Using NIH ImageJ, collagen fiber straightness was quantitated as the ratio of the length of a collagen fiber bundle (*L*_*f*_) to a straight line connecting the ends of the measured bundle (*L*_*s*_), where the lower the ratio of *L*_*s*_*/L*_*f*_ the wavier the fiber bundles, as described in Rezakhaniha *et al.*^27^ In graphs **a**, **c**, and **d**, ISC:MSC groups are not statistically different than uninjured skin. Asterisks show statistical difference from control wounds. Error bars show standard error of mean. Unfilled square control, yellow filled square MSCs, red filled square ISCs, blue filled square ISC:MSC, black filled square uninjured.

Hematoxylin and eosin (H&E) stained skin sections showed that ISC:MSC hydrogels accelerated the return of the structural layers of skin (Figs. 4, 5). Only uninjured skin and ISC:MSC-treated wounds had epidermal, dermal, and hypodermal layers. Wounds treated with ISC or MSC-only hydrogels developed epidermal and dermal layers, but not hypodermal layers. Untreated control wounds had not healed and showed few anatomical features of skin. Grades from slides evaluated by a pathologist were analyzed and ISC:MSC-treated wounds had the same number of ulcers as uninjured skin (zero), while all other treatments revealed ulcers (Fig. 6a).

Alpha smooth muscle actin (α-SMA) staining gave a measure of contraction occurring during wound healing (Fig. 5). Stains for platelet endothelial cell adhesion molecule (PECAM-1), also known as cluster of differentiation 31 (CD31), distinguished wound contraction from blood vessels, which also contain α-SMA. In uninjured skin, positive α-SMA stain was only present in the epidermis, surrounding sebaceous and sweat glands or vessel lumen. In wounded skin, positive α-SMA stain was present throughout tissue sections. In ISC:MSC wounds, positive α-SMA stain (brown) was predominately concentrated towards the epidermis, with less stain deeper in the dermis. In ISC treated wounds, positive α-SMA stain was also concentrated in the epidermis, with bands also appearing in the center of the dermis. In MSC treated wounds, positive α-SMA stain was equally present throughout the dermis and epidermis, suggestive of wound contraction. Diffuse α-SMA stain was visible in control wounds. Positive α-SMA staining was also detected surrounding vessel lumen with visible blood cells. Quantified blood vessel density (Fig. 6b) revealed that healing skin had greater numbers of CD31 stained vessel endothelia. CD31 staining specifically stains developing or mature vessel endothelia. Control wounds had not yet healed to the point to form vessels. Uninjured skin had few stained vessels, ISC:MSC had more, followed by MSC and ISC treated wounds, which had the most. Many tiny endothelia were observed in ISC or MSC treated wounds, representative of neovascularization and vascular proliferation in the granulation tissue.

Sections stained with the cellular marker for proliferation, Ki67, were quantified and treated wounds were compared to controls. All treated wounds had significantly more proliferating cells than controls, which had the least. Uninjured skin had the most migrating cells, followed by ISC:MSC, ISC, then MSC treated wounds (Fig. 6c) had the closest number of proliferating cells as in uninjured skin. Polarized picric acid sirius red (PASR) stained sections revealed the structure and relative content of collagen I and collagen III in skin explants (Fig. 6d). Uninjured skin had a basket-weave like collagen fiber arrangement, while collagen from wounds were more parallel. ISC:MSC-treated wounds showed waviness suggestive of the beginnings of a basket-weave like collagen arrangement. This observation was quantified (Fig. 6e) as described in Rezakhaniha *et al.*^27^ Collagen fiber bundles were measured at random locations within images with NIH ImageJ to obtain their lengths (*L*_*f*_), as was a straight line connecting the ends of the measured bundle (*L*_*s*_). Collagen fiber straightness was quantitated as the ratio of *L*_*s*_*/L*_*f*_ and returned values between 0 and 1, where values closer to 1 are straighter and those closer to 0 are wavier. Though not statistically distinct, uninjured skin was the waviest, followed by ISC:MSC treated groups. When collagen I and III ratios were quantified, ISC:MSC values were identical to that of uninjured skin.

## Discussion

For the first time, this study showed that the combination of ISCs and MSCs can accelerate slowed diabetic wound healing almost three times faster than these wounds would otherwise heal on their own (14 vs. ~ 40 days). We previously followed these chronic wounds to complete closure without treatment, which took 35–40 days.^1^ In fact, when treated with both ISCs and MSCs, these wounds closed even faster than wounds created in normoglycemic mice, where the healing time for this type of wound is 21–23 days.^12^ These surprising results raise several questions as to how the cells are achieving such closure. Particularly since MSC functional activity is highly regulated by matrix interaction.^21^ Yet these PEGDA hydrogels presented a bioinert environment and cells remained balled. Thus, a first question would interrogate why MSC viability remained high. One possible explanation is that MSC factor release increased simply as a result of encapsulation, and the release of MSC factors can have an autocrine effect on the MSCs themselves, improving their viability. Additionally, in other studies we have demonstrated the ability of MSCs to lay down matrix within these hydrogels,^19^ likely contributing to their long term viability.

In explaining the improved wound healing, one possibility is that the MSCs are differentiating into ISCs, and the hydrogels ultimately have twice the number of ISCs as were accounted for. This is unlikely for several reasons. First, following coencapsulation the release of MSC growth factors increased rather than decreased. Secondly, if the MSCs were differentiating to ISCs it would be expected that the ISC formulation (which had twice the number of ISCs as the ISC:MSC formulation) would improve healing at similar rates. However, although ISC:MSC hydrogels contained half the MSCs as MSC hydrogels and half the ISCs as ISC hydrogels, ISC:MSC hydrogels closed wounds twice as fast as either single cell hydrogel (14 vs. 28 days).

This finding was confirmed by histology: in ISC:MSC treated wounds, the Ki67 positive cells were mostly concentrated in the stratum basale while in ISC and MSC treated wounds, positive staining was visible in both the stratum basale and the dermis. Additionally, in normal wound healing, there is a localized increase in vascularity within the healing tissue. This localized vascularity recedes as the wound closes and matures. There were many small vessels in healing skin and fewer vessels were observed in uninjured and ISC:MSC-treated wounds, which is consistent with more mature skin.^34^ Taking these results together, the presence of multiple tiny blood vessels (angioplasia) and Ki67 positive cells in the dermal layer (fibroplasia) in ISC and MSC treated wounds suggests that these wounds were in the proliferative phase 34 while ISC:MSC treated wounds had progressed beyond it. Thus, the combination of the cell types was necessary for the accelerated 14-day wound healing.

Excluding MSC differentiation as a mechanism for the improved healing, another possible mechanism is that the system cured the animals of diabetes by delivering insulin, and thus in that way mitigating the wound healing deficits common to diabetics. Indeed, the combination of ISCs and MSCs has been used to treat diabetes for over a decade; this study is the first to use this combination for wound healing. The results do not indicate that diabetes was cured in the animals. Our system did not alter the weight nor the blood glucose levels of mice, who remained diabetic and obese. Thus, the accelerated wound healing was not a result of curing the diabetes but a result of the wound healing actions of the cells.

Although the possibilities that the MSCs differentiated into ISCs or the ISCs cured the mice of diabetes likely do not explain the results, there are several mechanisms that may explain the improved healing. First, the improved healing may be a result of the increased functionality and viability of both cell types. In fact, MSCs have been demonstrated to improve ISC viability and function.^6^,^36^ When combined with MSCs, islets were shielded from immune attack, increasing their function such that less islets were required to cure diabetic rats.^6^,^36^ Additionally, MSCs increased VEGF secretion when coenapsulated with ISCs, which promoted vascularization of ISC grafts.^6^,^36^ Our results confirm these findings. MSCs increased ISC insulin secretion by as much as threefold compared to ISCs encapsulated alone, and the insulin concentration in the media continued to increase until at least day 21. This increase in insulin release over time may be due to MSC actions to sustain ISC viability and improve their insulin secretion. For instance, TGF-β1 secretion increased with increasing ISC density (pilot data not shown), and TGF-β1 is known to preserve and prolong islet survival.^15^,^16^ Thus both cell types increased their secreted products in the presence of the other, which may explain the improved healing observed if such healing follows a dose response.

Interestingly, while the mechanisms for MSCs improving ISC viability and function has been reported, little has been reported on the reverse—the influence of ISCs on MSCs. Our findings showed increased viability and growth factor release from coencapsulated MSCs, suggesting that ISCs improve the function and viability of MSCs. For instance, MSCs cultured alone released little to no VEGF and TGF-β1 *in vitro.* When ISCs were present, MSC release of these factors increased significantly. One possible mechanism for these increases may be insulin’s action on the PI3K-Akt/PKB pathway. To mitigate the apoptosis that most transplanted MSCs undergo shortly following transplantation,^4^ groups have subjected MSCs to hypoxic preconditioning, which improves their viability and increases their expression of growth factors and cytokines upon transplantation 11,13 by activating the PI3K-Akt/PKB pathways.^4^,^11^ Whether through hypoxic conditioning or genetic modification, activating the PI3K-Akt/PKB pathway has consistently resulted in increased MSC expression of growth factors, cytokines, and anti-apoptotic effects have been observed.^4^,^9^,^11^,^13^,^20^ Importantly, insulin also activates this pathway.^14^ Thus, the improved MSC viability and function we observed when MSCs were combined with ISCs is likely due to the activation of the PI3K-Akt/PKB pathway. Hence, while it was known that MSCs improve ISC fate, for the first time we show that in return, ISCs improve MSC viability and function.

In addition to supporting MSC function, insulin likely protects MSC growth factors by mediating the protease rich environment of the chronic wound; insulin is a key player in regulating extracellular proteolytic pathways.^32^ In addition, insulin stimulates the synthesis of a wide variety of matrix proteins,^2^,^8^,^23^,^30^ and it has been posited that insulin’s actions to reduce catabolism while also increasing matrix deposition contributes to improved wound healing.^17^

The increased presence of MSC factors may also explain the absence of scars—none of the wounds treated with ISC:MSC hydrogels developed scabs or scars. Histological findings supported these visual observations. Type I and III collagens play a critical role in scar formation; though necessary for wound healing, an excess deposition of collagen can result in scarring.^35^ Therefore, a critical balance of type I and III collagen is required for scar-free healing.^22^ The collagen in ISC:MSC-treated wounds was less densely packed than other wounds, and the collagen I/III ratio was identical to that of uninjured skin. Conversely, scars have highly densely packed collagen with a lower collagen I/III ratio than normal skin,^38^ strongly supporting our visible observation that ISC:MSC wounds healed without scar. Histology of day 14 vs. day 28 harvests showed continued remodeling, suggesting it would continue towards normal skin rather than scar. This absence of scar has been shown using MSCs derived from Human Wharton’s Jelly (WJ-MSCs).^29^ The authors compared the healing capacity of these fetal WJ-MSCs to adult bone marrow derived MSCs and found that the fetal MSCs were capable of augmenting scar-free wound healing. They concluded that the cause was due to the increased release of growth factors from the WJ-MSCs over the adult MSCs. Our results show that when combined with ISCs, MSCs increase their release of growth factors over MSCs that are not co-encapsulated. As the WJ-MSCs increased growth factor release contributed to scar free healing, likewise, the increased release and persistence of growth factors due to the action of insulin is the most likely actor in the scar-free healing observed herein.

## Conclusions

We have for the first time demonstrated dramatically accelerated healing using a dual cell therapy technique with ISCs and MSCs. Should these results be translatable to humans, the accelerated healing we observed could transform the treatment of a variety of difficult wounds, including chronic and diabetic wounds, and thermal and radiation burns, the latter of which has no standard of care. The scar-free healing we observed could revolutionize reconstructive surgery by reducing or eliminating scar formation, thus improving functional and cosmetic outcomes in plastic surgery or for those who suffer from hypertrophic scar. Ultimately, lessons learned from this system may pave the way for future dual cell therapies.

## Abbreviations

ANOVA: Analysis of variance
α-MEM: Alpha-minimal essential medium
α-SMA: Alpha smooth muscle actin
bFGF: Basic fibroblast growth factor
CD31: Cluster of differentiation
EGF: Epidermal growth factor
ERK: Extracellular signal-regulated protein kinase
FBS: Fetal bovine serum
H&E: Hematoxylin and eosin
IACUC: Institutional animal care and use committee
ISC: Insulin secreting cells
MSC: Mesenchymal stem cells
PASR: Picric acid sirius red
PDGF: Platelet derived growth factor
PECAM-1: Platelet endothelial cell adhesion molecule
TGF-β1: Transforming growth factor β1
VEGF: Vascular endothelial growth factor
